# Fluorescence-Guided Surgery and Fluorescence Laparoscopy for Gastrointestinal Cancers in Clinically-Relevant Mouse Models

**DOI:** 10.1155/2013/290634

**Published:** 2013-02-19

**Authors:** Cristina A. Metildi, Robert M. Hoffman, Michael Bouvet

**Affiliations:** ^1^Department of Surgery, University of California, San Diego, CA, USA; ^2^AntiCancer, Inc., San Diego, CA, USA; ^3^Moores UCSD Cancer Center, 3855 Health Science Drive No. 0987, La Jolla, CA 92093-0987, USA

## Abstract

There are many challenges that face surgeons when attempting curative resection for gastrointestinal cancers. The ability to properly delineate tumor margins for complete resection is of utmost importance in achieving cure and giving the patient the best chance of prolonged survival. Targeted tumor imaging techniques have gained significant interest in recent years to enable better identification of tumor lesions to improve diagnosis and treatment of cancer from preoperative staging modalities to optimizing the surgeon's ability to visualize tumor margins at the initial operation. Using unique characteristics of the tumor to fluorescently label the tissue can delineate tumor margins from normal surrounding tissue, allowing improved precision of surgical resection. In this paper, different methods of fluorescently labeling native tumor are discussed as well as the development of fluorescence laparoscopy and the potential role for fluorescence-guided surgery in the treatment of gastrointestinal cancers.

## 1. Introduction

The primary treatment modality for most patients with solid tumors is surgery. There are a multitude of factors that can significantly alter a patient's postoperative survival, such as tumor size, histological tumor grade, and vessel invasion [6, 7]. However, it is lymph node status and a complete surgical resection (R0 resection) that provides the patient with the most valuable prognostic information with regard to postoperative survival [6, 8]. 

Targeted tumor imaging techniques have gained significant interest in recent years to enable better identification of tumor lesions to improve diagnosis and treatment of cancer, from preoperative staging modalities [9–12] to optimizing the surgeon's ability to visualize tumor margins at the initial operation [1, 2, 13–19]. Using unique characteristics of the tumor to fluorescently label the tissue can delineate a margin between tumor and adjacent tissue, allowing improved precision of surgical resection. One example is the use of activatable probes that rely on high tumor tissue enzymatic activity [17]. Other examples include using fluorophore-conjugated antibodies to unique surface markers expressed by individual tumor types [14, 16], or the use of replication-competent viruses engineered to express the green fluorescent protein (GFP) in the presence of activated telomerase [10, 15]. In this review, we will discuss the progression of fluorescence-guided surgery and laparoscopy as well as its future directions and its potential use in the clinical treatment of gastrointestinal cancers. 

## 2. Development of Fluorescence Imaging Prototypes and Applications

 Herpes simplex-1 virus, NV1066, a replication-competent virus was engineered to infect and lyse cancer cells selectively. In addition, the virus contained a transgene for green fluorescent protein (GFP) that would result in fluorescent cells upon infection. *In vivo* infection of NV1066 resulted in localized expression of GFP to the tumor, which could be visualized endoscopically with the use of a laparoscope and appropriate fluorescence filters. Furthermore, the NV1066 selectively infected and replicated within the esophageal cancer cells, killing the cells *in vitro* and *in vivo *[20].

NV1066 was used to infect mouse models with lymphatic metastasis of human mesothelioma cancer cells. NV1066 injected into primary tumors was able to locate and infect lymph node metastases, producing GFP-expressing metastases which were easily visualized under fluorescence imaging. The fluorescence thoracoscopy model used in this experiment [21] involved an excitation filter on the light source set at 470 ± 20 nm and an emission filter on a camera processor set at 510 nm.

Tumors were also selectively and accurately labeled with GFP using a telomerase-dependent adenovirus (OBP-401) containing the GFP gene [15, 22, 23] and subsequently resected under fluorescence guidance. Recurring cancer cells maintained GFP expression after fluorescence-guided surgery, enabling the detection of recurrence and future metastasis possible with OBP-401 GFP labeling [22]. Maintenance of label in recurrent tumors is not possible with nongenetic probes. 

## 3. Development of Fluorescence Laparoscopy

 With new techniques emerging to fluorescently label tumors, fluorescence laparoscopy is becoming an exciting field of investigation. An optimal fluorescence laparoscopy model should maximize the fluorescence signal of the tumor for easy and rapid imaging and also provide adequate background illumination to visualize surrounding tissues to allow for spatial orientation without compromising the tumor-to-background contrast.

**Figure 1:**
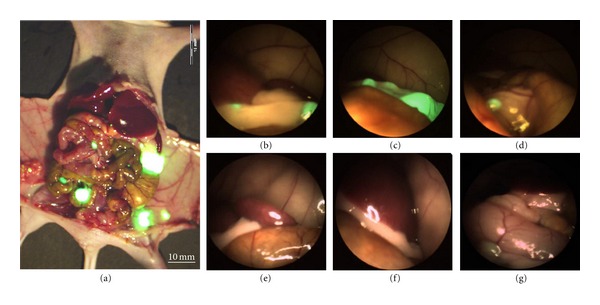
Comparative identification of tumor foci under brightfield and fluorescence laparoscopy. (a) OV100 open image from a representative mouse. View of left upper quadrant in a mouse specimen under FL (b–d) and BL (e–g). The green fluorescence of the metastatic lesions are unmistakable under FL, whereas under BL the tumor foci resembled normal tissue and were not identifiable. BL: brightfield laparoscopy; FL: fluorescence laparoscopy [1].

Our group developed a fluorescence laparoscopy model with the use of a Xenon light source that permitted facile, real-time imaging and localization of tumors labeled with fluorescent proteins within the abdomen of a mouse [18]. A standard laparoscopic system was easily modified by placing a 480 nm short-pass excitation filter between the light cable and the laparoscope. A 2 mm-thick emission filter was placed between the laparoscope and camera. The use of proper filters enabled simultaneous visualization of fluorescent tumor and non-fluorescent normal tissue and greatly enhanced the diagnostic capabilities of staging laparoscopy (Figure 1) [1].

**Figure 2:**
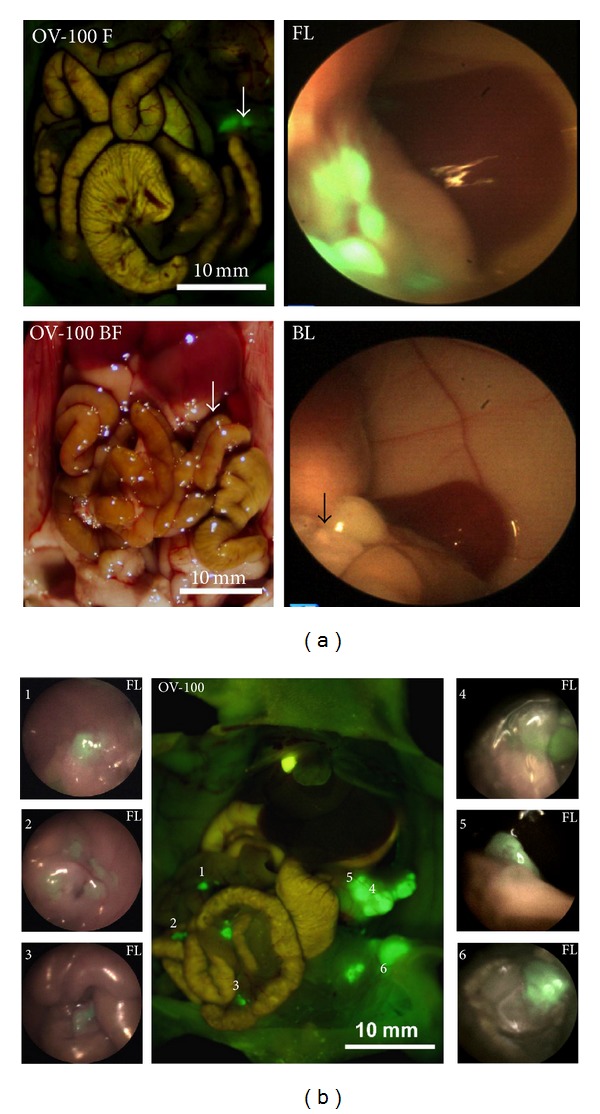
(a) Fluorescence laparoscopy with fluorophore-conjugated antibodies. Images under fluorescence and bright field laparoscopy visualized the primary tumor in the body of the pancreas. The two images on the left are positive control images taken with the Olympus OV-100 small animal imaging system for comparison with laparoscopic images on the right under fluorescence (top) and bright field (bottom). The primary pancreatic tumor was more easily detected under fluorescence laparoscopy (FL) compared to bright field laparoscopy (BL) [2]. (b) Use of fluorescence laparoscopy to identify primary and metastatic lesions. The center image is a positive control OV-100 image for comparison with BL and FL. The surrounding images, labeled 1–6, are representative FL images of primary and metastatic pancreatic tumor lesions. The numbers in the upper left corner of each picture correspond to the numbered lesion in the center OV-100 image [2].

Fluorophore-conjugated antibodies directed at unique tumor antigens were also used to fluorescently label tumor [2]. Kaushal et al. [14, 16] used antibodies directed against common tumor antigens to deliver fluorophores for enhanced detection of tumors during laparotomy in orthotopic mouse models of pancreatic and colon cancer. Fluorescence laparoscopy significantly enhanced the sensitivity and positive predictive value of diagnostic laparoscopy. Tumor detection was quicker and more accurate with very few false positives (Figure 2).

**Figure 3:**
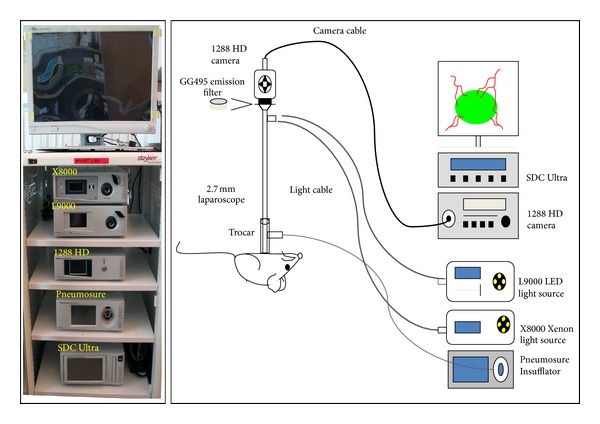
Stryker laparoscopic setup. A standard laparoscopic tower was slightly modified to achieve a fluorescence light mode that permitted detection of fluorescence signals while still allowing visualization of the background. The LED light source (Stryker L9000 LED lamp) was filtered through a glass emission filter (Schott GG495) that was placed between the laparoscope and the 1288 HD camera. With alterations to red, blue, and green components of the light source, we were able to visualize tumors of different fluorescent wavelengths. A Stryker X8000 Xenon light source was used for bright field laparoscopy [3].

**Figure 4:**
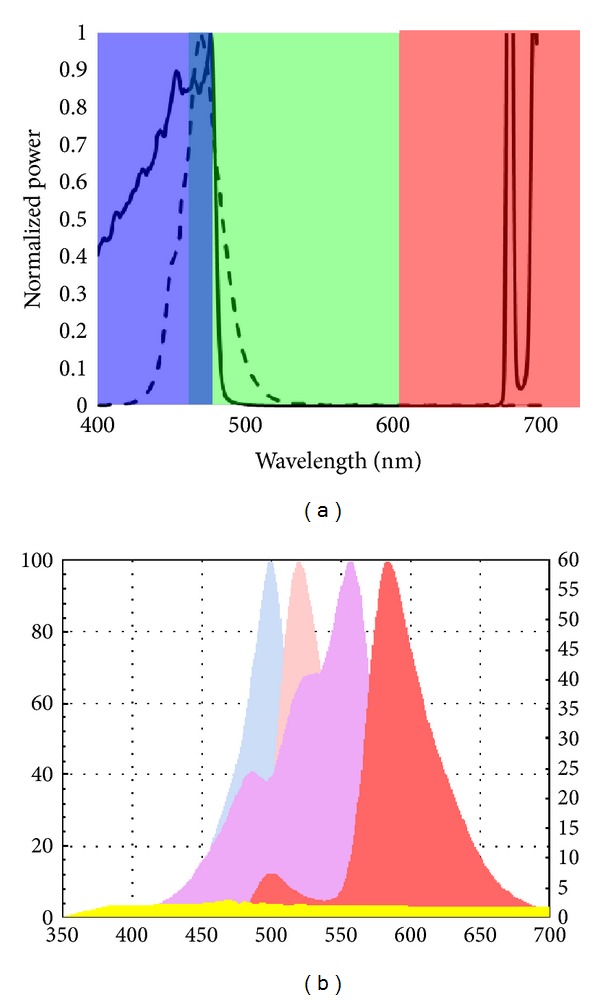
(a) The spectrum of two possible light sources for fluorescence guided surgery. The solid line illustrates the spectrum of a filtered Xenon lamp as used in Tran Cao et al. [1]. The dash line illustrates a typical blue LED spectrum. The color blocks mark the spectral range of red, green, and blue channels on common RGB CCDs. (b) Overlapping emission and excitation spectra of GFP/Alexa-488 and RFP/Alexa-555 fluorescent proteins and fluorophores. Blue and salmon color peaks represent the excitation and emission spectra of GFP/Alexa 488, respectively. Pink and Red peaks represent excitation and emission spectra of RFP/Alexa 555, respectively. This graphic demonstrates the utility of the overlapping spectra of these fluorophores in the spectral range of GFP in detecting tumor while maintaining adequate visualization of surrounding structures for spatial orientation and surgical navigation. Filtering an LED light source through a 495 glass filter creates the bandwidth by which tumors of different fluorescent colors are visualized simultaneously [3].

**Figure 5:**
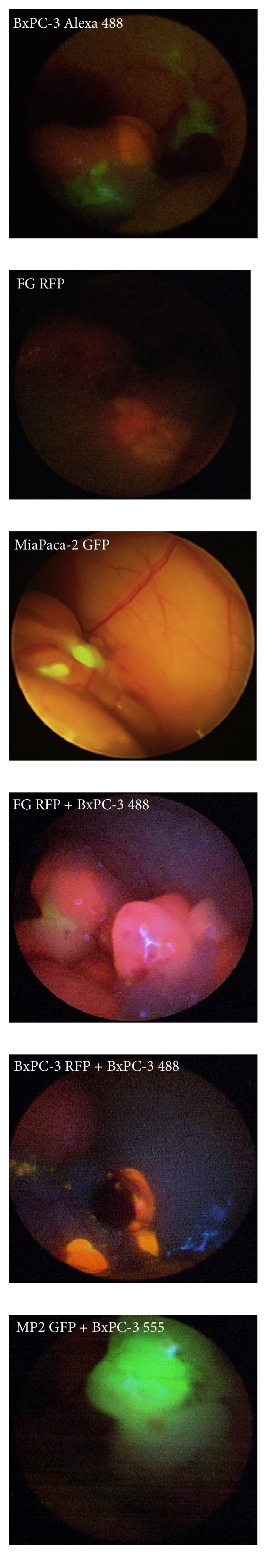
Laparoscopic images of the left upper quadrant in representative mouse models of human pancreatic cancer labeled with fluorophores with different fluorescence wavelengths. Fluorescence laparoscopy with the LED light source allows identification and localization of human pancreatic tumors of different fluorescence wavelengths simultaneously with improved accuracy. The combination of RFP-expressing tumor labeled with AntiCEA Alexa 488 afforded the brightest signal [3].

However, due to the lack of intensity from the filtered Xenon light, adjustments to exposure time and gain were necessary. However, increasing the exposure time and gain resulted in significant dynamic delay that impaired surgical navigation. Replacing the Xenon light source with an LED lamp virtually eliminated the need for an excitation filter between the light cable and laparoscope (Figure 3) [3]. With only the use of an 495 nm emission filter along with adjustments to the red, blue, and green components of the LED, no adjustments to exposure time or gain was necessary, and rapid detection of fluorescent tumor was greatly improved while also allowing visualization of surrounding tissue which can enable surgical navigation (Figure 4). This new model of fluorescence laparoscopy, with maximal blue light and adjustments to red and green light, produced a spectrum of light transmission that resulted in proper color balance and adequate background illumination. This enhanced the fluorescence signal-to-background ratio, enabling real-time simultaneous detection of tumors with different fluorescent colors (Figure 5). 

**Figure 6:**
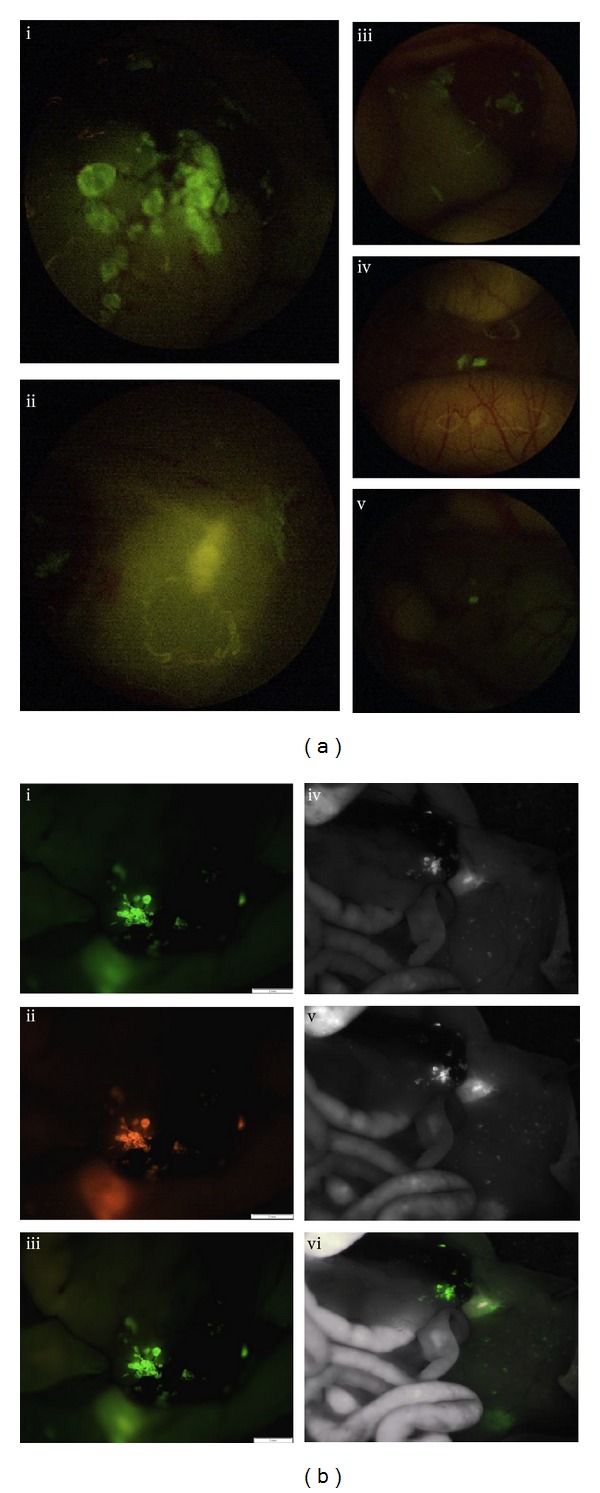
Orthotopic mouse model of BxPC-3 human pancreatic cancer dual labeled with anti-CEA Alexa 488 and 555. (a) Laparoscopic images of representative mouse specimen with BxPC-3 dual labeled with anti-CEA Alexa 488 and 555. The combination of red and green fluorophores creates a significantly brighter fluorescence signal without compromising background illumination. (i–iii) are laparoscopic images of the left upper quadrant. (iv) and (v) are laparoscopic images of metastatic tumor deposits hidden within the mesentery of the mouse. These deposits were virtually undetectable under BL. (b) (i–iii) are intravital OV-100 images of the same mouse specimen under (i) GFPa filter (excitation 460–490; emission 510–550), (ii) RFP filter (excitation BP 535–555; emission 570–623), and (iii) GFP (excitation 460–490; emission 510F) filters. The bottom image (iii) corresponds to GFP bandwidth through which fluorescence laparoscopy is viewed. (iv–vi) are the corresponding intravital Maestro images. (iv) and (v) are spectral unmixing images of the (vi) compositive image obtained through (iv) GFP and (v) RFP filter sets, respectively. These images confirm the dual labeling of BxPC-3 tumor with Alexa 488 and 555 [3].

The ability to visualize differently fluorescent tumors simultaneously resulted in the identification of an optimal fluorophore for fluorescence laparoscopy [3]. The combination of dually labeling nonfluorescent tumor with Alexa 488 and Alexa 555 greatly enhanced the fluorescence signal allowing for better detection of sub-millimeter deposits throughout the abdomen (Figure 6). The combination of red and green fluorophores optimized the fluorescence signal of tumor allowing accurate distinction of tumor margins without compromising background illumination. This permitted laparoscopic resection of tumors in mouse models of pancreatic cancer. The improved visualization of surrounding structures for surgical navigation without compromising tumor detection further demonstrates the potential therapeutic uses of fluorescence laparoscopy. 

## 4. Fluorescently Labeling Native Tumors 

 In addition to using fluorophore-conjugated antibodies and GFP-containing viruses, there have been a variety of methods described to fluorescently label native tumor. 

Activatable cell penetrating peptides (ACPPs) have been used as targeting agents for cancer cells. Polycationic cell penetrating peptides (CPPs) are connected via a cleavable linker to a neutralizing polyanion whose adsorption and uptake into cells are inhibited until the linker is proteolyzed. With the upregulation of MMP-2 and MMP-9 in most solid tumors, exposure to these proteases results in cleavage and dissociation of the inhibitory peptide, allowing the CPP to bind to and enter cancer cells. Conjugating CPPs to a fluorophore then enables improved visualization of the tumor. Further conjugating dendrimers to ACPPs (ACPPDs) results in a higher absolute tumor fluorescence and tumor-to-background fluorescence contrast than free ACPPs [17]. 

## 5. Future Directions of Fluorescence-Guided Surgery and Laparoscopy 

**Figure 7:**
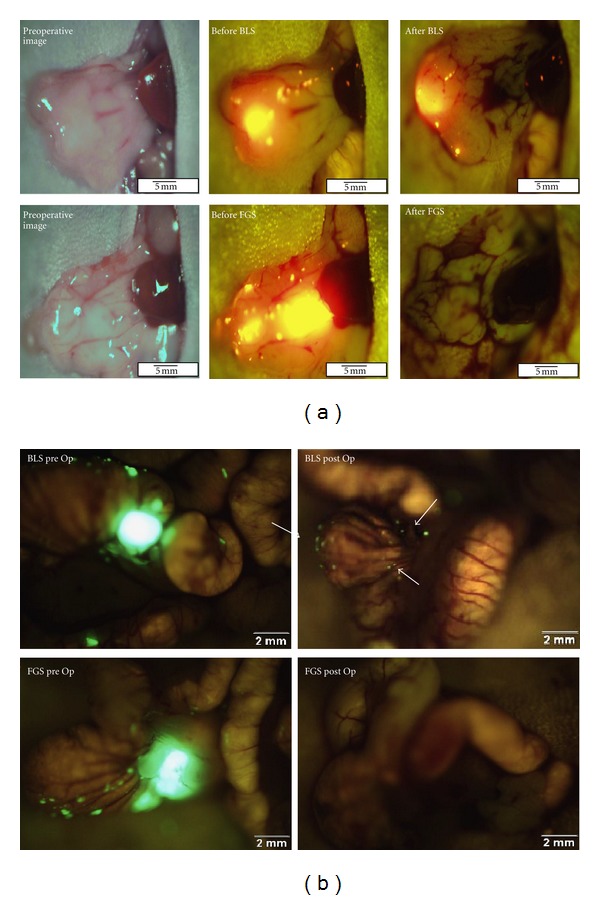
Surgical resection after fluorescence-guided surgery (FGS). (a) The top row are representative pre- and postoperative images of a mouse specimen from the bright-field surgery (BLS) group. The tumors were labeled with RFP. A tumor reduction of only 77% was achieved in the BLS group. The bottom row images are representative pre- and postoperative images of a mouse specimen from the FGS group. A significant improvement in tumor reduction was achieved in this group (98.9%, *P* = 0.005). A complete surgical resection of pancreatic tumor with negative surgical margins was achieved in this mouse without requiring significant resection of the pancreas. (b) Representative pre- and postoperative images of a mouse from the BLS group (top panel) and the FGS group (bottom panel). The enhanced ability to visualize and identify tumor margins under fluorescence-guidance permitted a more complete resection. The tumors were labeled with GFP. All mice in the FGS group underwent an R0 resection while only 58% of mice in the BLS group had no evidence of residual fluorescent tumor on postoperative images (arrows in right upper panel) (*P* = 0.001) [4, 5].

Our recent work with fluorophore-conjugated antibodies (FCAs) directed against the tumor antigen CEA has shown to be a method of labeling, detecting and subsequently resecting tumor to improve surgical outcomes in mouse models of pancreatic and colon cancer [3, 14]. The significant improvement in resection of primary tumor lesions achieved under fluorescence-guided surgery significantly reduces the postoperative tumor burden in mouse models of human cancer (Figure 7). Furthermore, the greater incidence of achieving an R0 resection in these mouse models results in longer disease-free survival and overall survival. 

 The goal is to improve methods of fluorescently labeling native tumor to permit better preoperative detection of metastatic tumor and to further enhance the surgeon's ability to delineate tumor margins and allow more objective means of identifying and resecting all tumor at the initial operation.
